# The impact of very preterm vs very low birth weight on early and mid-adulthood preference-based HRQoL outcomes: findings from the Dutch study on preterm and small for gestational age infants

**DOI:** 10.1007/s11136-025-04024-8

**Published:** 2025-09-13

**Authors:** Corneliu Bolbocean, Paula van Dommelen, Stephen O’Neill, Sylvia van der Pal

**Affiliations:** 1 Nuffield Department of Population Health Care Sciences, University of Oxford, Oxford, UK; 2 Netherlands Organisation for Applied Scientific Research, Leiden, The Netherlands; 3 London School of Hygiene and Tropical Medicine, London, UK

**Keywords:** HUI3, SF-6D, HRQoL, Health utilities, Very preterm birth, Very low birth weight

## Abstract

**Objectives:**

Very preterm (VP, < 32 weeks gestation) birth and very low birth weight (VLBW, < 1500 g) are distinct but overlapping risk factors with different clinical implications. We aimed to investigate the separate and combined impacts of being born VP and/or VLBW on health-related quality of life in early and mid-adulthood.

**Methods:**

We analyzed data from the Dutch Project on Preterm and Small-for-gestational-age infants (POPS), a national prospective cohort of individuals born in 1983. Participants were categorized into three groups: (1) VP & VLBW, (2) VP-only, and (3) VLBW-only. We used the Health Utilities Index Mark 3 at ages 19 and 28, and the Short Form 6-Dimension at age 35 to assess multi-attribute utility (MAU) scores and domain-level functioning. Adjusted linear regression models were used, controlling for covariates and employing inverse probability weighting to account for attrition.

**Results:**

Overall MAU scores did not consistently differ between the exposure groups and the VP & VLBW reference group at any time point. However, specific domain-level differences emerged in early adulthood. At 19 years, the VLBW-only group reported significantly better speech functioning (β = 0.11, p = 0.01). At 28 years, the VP-only group had better hearing (β = 0.05, p = 0.04), while the VLBW-only group had worse ambulation (β =  − 0.12, p < 0.01). By 35 years, these inter-group differences were no longer statistically significant. Female sex was a consistent predictor of poorer outcomes in several domains by age 35. Attrition-weighted models produced nearly identical results.

**Conclusions:**

VP and VLBW are not interchangeable risk categories. While overall HRQoL scores converged by mid-adulthood, distinct domain-specific and sex-based disparities were evident earlier in life. Our findings highlight the need for tailored interventions over a homogenous approach. Future research with consistent measures is required to confirm if this convergence persists over the life course.

**Supplementary Information:**

The online version contains supplementary material available at 10.1007/s11136-025-04024-8.

## Plain English Summary

1. *Why is this study needed?* Babies born very early (before 32 weeks of pregnancy) or with a very low birth weight (less than 1,500 grams) can have health challenges later in life. However, it's not well understood if these two conditions—being born too early versus being born too small—affect a person's quality of life differently as they grow up.
2. *What is the key problem this study addresses?* This study examines whether the long-term impact on overall well-being is different for adults who were born very early, very small, or a combination of both.
3. *What is the main point of your study?* We followed a group of adults who were born in the Netherlands in 1983 and tracked their health-related quality of life at ages 19, 28, and 35 to see how being born very early and/or very small affected overall quality of life.
4. *What are your main results and what do they mean?* The main finding is that while the overall quality of life for all groups was similar by age 35, there were important differences in specific areas like speech, hearing, and walking ability in their teens and twenties. This means that being born "very early" and being born "very small" are distinct challenges. Therefore, support and follow-up care should be tailored to the specific needs of each group, rather than treating all individuals born prematurely the same.

## Introduction

Very premature (VP) births, defined as those with a gestational age of less than 32 weeks and those with very low birth weight (VLBW) i.e. less than 1500 g, have consistently been associated with increased mortality risk [1–3], adverse neurodevelopmental outcomes [4–6], and substantial socio-economic challenges that persist into early to mid-adulthood [7–10]. At the same time, an alarming rise in preterm and VP birth rates and improved survival odds have amplified the financial strain on healthcare systems globally [11–13]. Consequently, prematurity has emerged as a pressing public health issue, necessitating detailed examination of the corresponding economic challenges to inform and shape evidence-based policies. To effectively evaluate and enhance these policies and interventions, health-related quality of life (HRQoL) measures are utilised. However, such data is often limited, primarily due to lack of longitudinal studies which track HRQoL over time [14].

Generic measures of HRQoL are comprehensive constructs [15, 16] that demonstrate robust correlations with widely used health indicators such as morbidity, mortality, and healthcare costs [17–19]. Specifically, HRQoL measures that are paired with preference -based value sets yield utility scores that depict health state preferences. Health utilities are instrumental in healthcare economic and policy assessments [20–22] and can provide insight into the daily effects of individual functioning when incorporated into clinical studies [23–26] and healthcare economic and policy evaluations [20–22]. There is a limited research which investigates the separate effects of being born VP or with VLBW on HRQoL. Specifically, research has only investigated the VP and VLBW distinct implications on clinical outcomes, as reported in the literature [27, 28] and has generally found that VP and VLBW can lead to different clinical outcomes. Thus, the implications of being born VP vs VLBW on overall well-being are not fully understood. A recent meta-analysis found evidence on the association between VP/VLBW status and HRQoL in adulthood using Health Utilities Index Mark 3 (HUI3) and not Short Form 6 (SF-6D) [29], however, this analysis did not clarify the distinct implications of being born either VP or with VLBW. Given the vital distinction between VP and VLBW, understanding their unique implications on HRQoL is crucial. Thus, while VP and VLBW often intersect, they do not always coincide. For instance, a full-term baby could still have VLBW due to conditions such as intrauterine growth restriction. Fetal growth restriction often operationalized as being small for gestational age can have wide-ranging biological and psychosocial implications that extend into adulthood [30]; may predispose individuals to long-term metabolic conditions or neurodevelopmental challenges. Socially, early health complications might restrict educational and employment opportunities or influencing income [31] underscoring how combined or separate effects of VP and VLBW might shape adult HRQoL.

This examination of differential of birthweight and gestational age is important because the use of previously reported utility values for preterm birth as a whole, ignoring the specific differences between VP and VLBW may lead to misleading cost-effectiveness estimations. A better understanding of these impacts can help in assessing the cost-effectiveness of interventions targeted at these populations ultimately improving HRQoL for these individuals. Overall, this can help provide better support to individuals affected by these conditions and enable cost-effectiveness-based decision-making.

In this study, we aimed to investigate the combined and separate impact of VP and/or VLBW on HRQoL using data from the Dutch Project on Preterm and Small-for-gestational-age infants (POPS). To our knowledge, POPS is the only study population based cohort that has recruited almost all infants born alive in 1983, VP and/or with VLBW in the Netherlands and followed them into adulthood. This study aimed to ascertain the impact of being both VP and VLBW compared with either alone.

## Methods

### Data

**Fig. 1:**
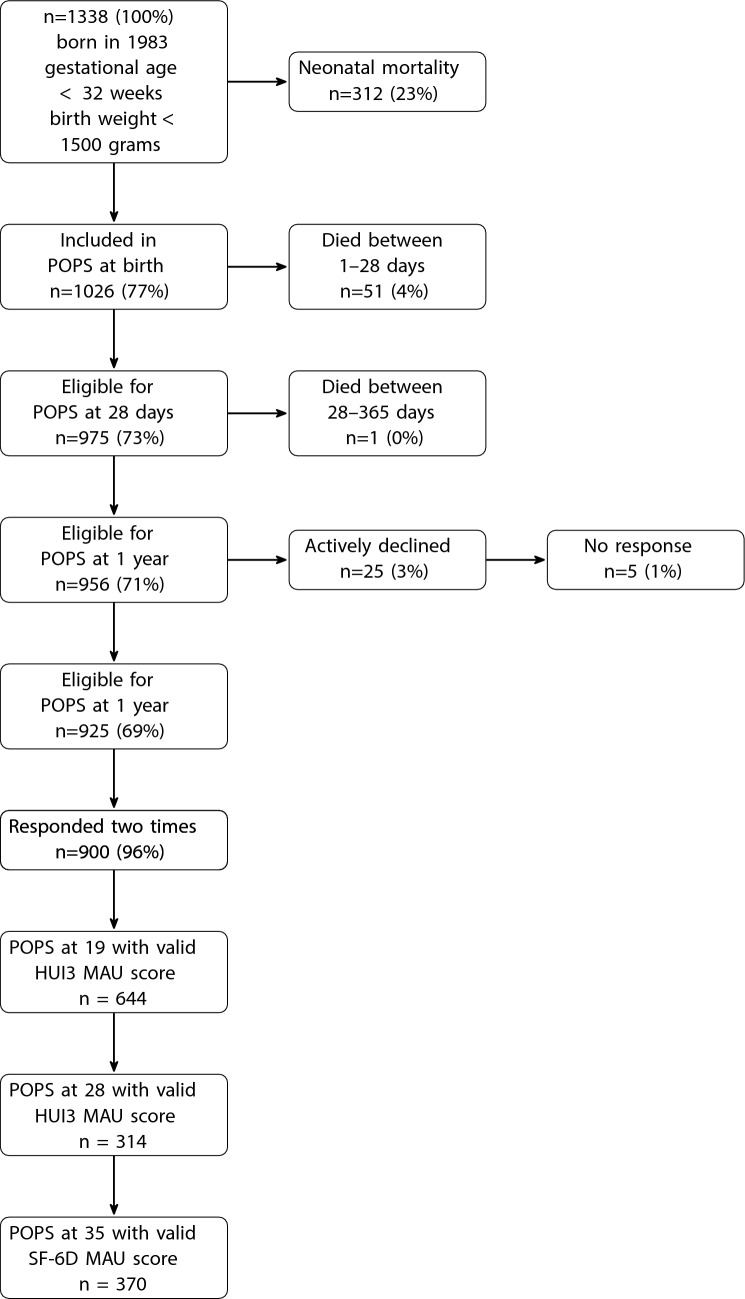
Flowchart of participants from birth through follow-up. The participant flow for this cohort has been previously described in Van Der Pal, S. M., et al. European Journal of Pediatrics 180.4 (2021): 1219–1228

The POPS cohort included 94% (n = 1338) of all live-born infants in the Netherlands in 1983 with a gestational age of less than 32 weeks (VP) and/or a birth weight below 1500 g (VLBW). Figure 1 shows the flowchart of participants from birth through follow-up. Although 1338 infants were originally enrolled and this number is reported in earlier publications [32], the analytic dataset comprised of 1336 unique participants. During data transfer, the POPS investigators discovered that one twin pair had been entered twice.

The inclusion criteria of the POPS study allow the comparison of three distinct groups: (1) infants who were born VP & VLBW (i.e. combined effect), (2) infants who were born VP-only, and (3) infants who were VLBW-only. Gestational age was determined using data from the last menstrual period, pregnancy tests, and/or ultrasound findings.

### Outcome variables

This study examined the HRQoL of individuals born VP or VLBW who reached 19, 28 or 35 years. Two preference-based measures have been used: Health Utility Index Mark 3 (HUI-3) and Short Form 6 Dimensions (SF-6D). The multi-attribute utility scores HUI3 (at 19 and 28 years) and SF-6D (at 35 years) are the main outcomes of interest. POPS study investigators decided to replace HUI3 at 35 with SF-12/SF-6D because of HUI3 licensing costs and questionnaire length constraints.

Study participants completed the HUI3 for usual health status assessment which comprises eight attributes (ambulation, dexterity, cognition, vision, hearing, speech, emotion, and pain). Algorithms reflecting the preferences of the general public for the HUI3 health states can convert these responses into multiplicative multi-attribute utility scores (HUI-MAU), and we applied the Canadian algorithms [33–36], with HUI3 multi-attribute utility scores valued between [− 0*.*36*,*1*.*0], where − 0.36 represents the worst possible HUI3 health state, 0.0 represents death, and 1.0 represents optimal health [33, 34]. The SF-12 includes 12 of the SF-36 items [37] yielding an eight-dimension profile scaled 0–100 converted into SF-6D utility scores [range (0, 1.0)] using UK algorithm [38].

### Main exposure

The main independent variables in this study were: an indicator for VP & VLBW, an indicator for VP-only (VP and not VLBW), and an indicator for VLBW-only (VLBW and not VP).

### Covariates

A number of socio-economic characteristics have been shown to correlate with HRQoL among preterm individuals [39, 40]. Thus, consistent with previous literature [39, 40], the following independent variables were included in the adjusted analysis [39, 40]: sex, age (in years) at assessment, and mother’s level of education at time of birth of the child and during childhood standardized according to the International Standard Classification of Education (ISCED) into low (ISCED levels 0–2), medium (ISCED levels 3–5), and high (ISCED levels 6–8) [41] (Low maternal education (reference) category in all models), maternal age and as well as maternal ethnicity. Education is used as a proxy for economic well-being [42, 43]. The literature shows that ethnicity [44, 45], and age [46] might serve as indirect proxies for socioeconomic factors.

## Statistical analysis

We plotted each individual’s birth weight against gestational age and overlaid cutoff lines representing 1500 g and 32 weeks respectively. To visualize cohort distributions, we used hexagonal bin diagrams in which color either indicated the number of subjects per bin or the bin-specific mean of a health utility measure (*HUI3* or *SF-6D*). Separate hex-bin plots for *HUI3* at 19 and 28, and *SF-6D* 35 years, allowed us to depict how average utility varied across different combinations of gestational age and birth weight. We calculated means, standard deviations and performed Analysis of Variance (ANOVA) for unequal variances, and medians to assess differences in the outcome measures between VP&VLBW, VP only and VLBW only.

We initially estimated the association between being born VP&VLBW, VP-only, or VLBW-only and HRQoL in adulthood. We used ordinary least squares to model the adjusted impacts of VLBW-only and VP-only relative to VP&VLBW on MAU scores; a linear probability model to model (LPM) the impact on optimal level of functioning. LPM offers easily interpretable percentage-point effects for binary outcomes. However, we used logistic models as robustness checks to examine the adjusted impact of being born VP&VLBW, VP-only, or VLBW-only on optimal level of functioning.

Models were adjusted for covariates described in “Covariates” section. We used inverse probability weighting (IPW) adjustment to account for potential bias due to attrition across different waves. In particular, we used the following covariates to predict attrition at 28 and at 35 years: age, sex, birthweight, gestational age, maternal ethnicity and maternal age at birth. Analyses were performed using STATA version 18 (Stata Corp, College Station, TX) and two-sided p-values of 0.05 or less were considered statistically significant.

## Results

### Baseline characteristics

**Fig. 2:**
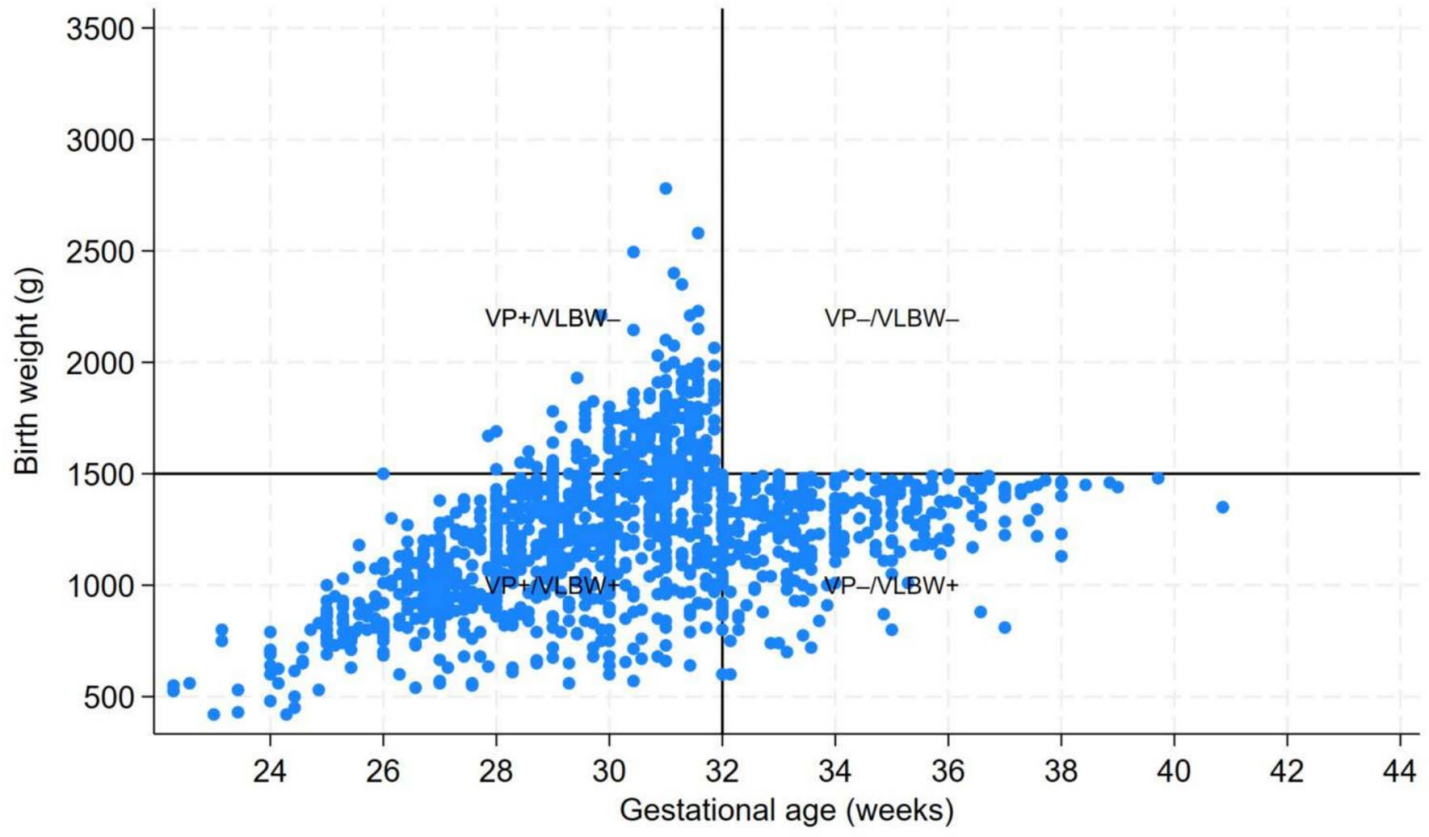
Distribution of POPS study population, with the three different groups indicated

**Fig. 3:**
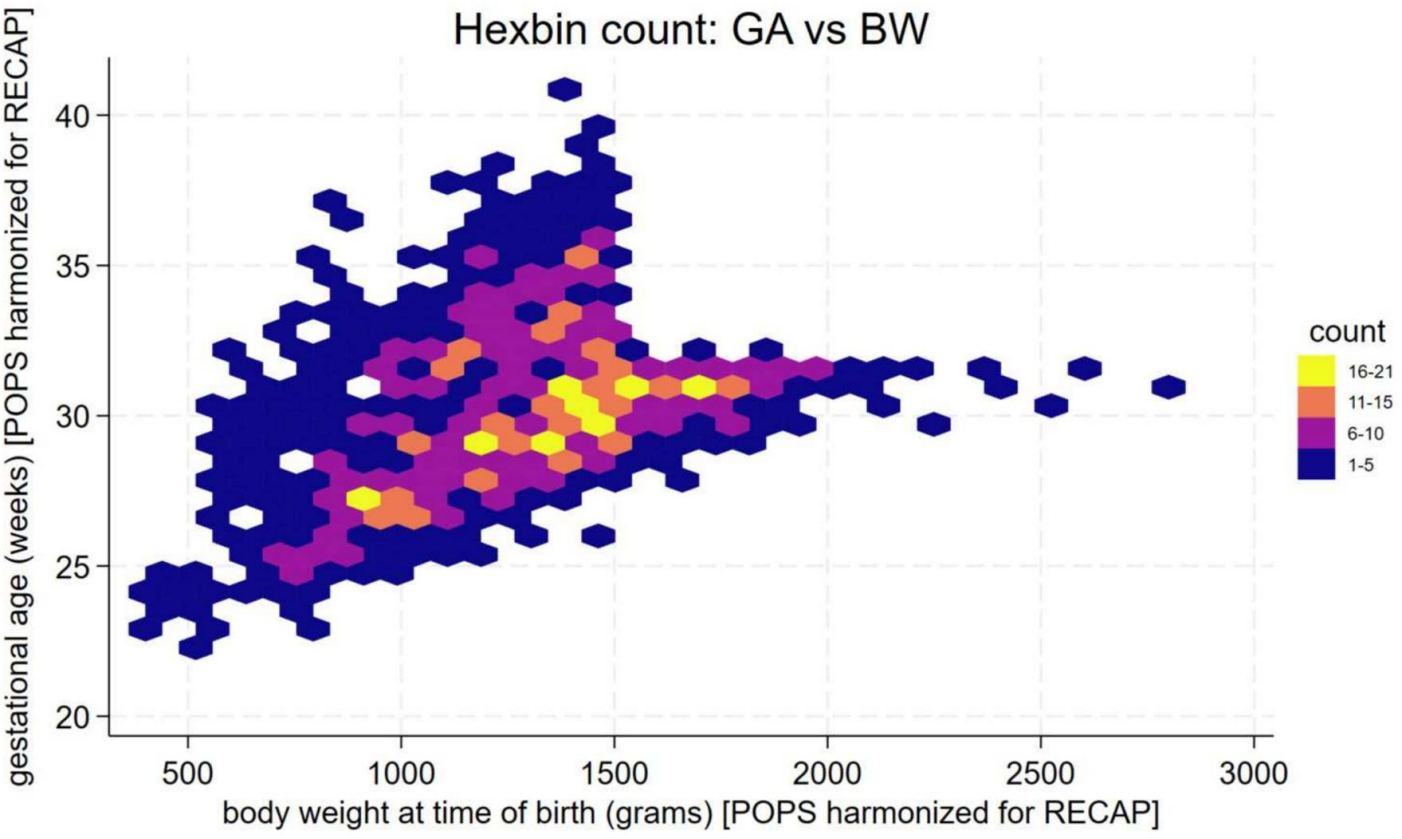
Distribution of POPS study population, with the three different groups indicated

In Fig. 2, we show the distribution of birth weight (vertical axis) by gestational age (horizontal axis) for infants classified into one of three groups based on whether they were VP and/or VLBW. Most infants cluster in the lower-left quadrant (VP & VLBW). A smaller subgroup lies above the 1500 g line but below 32 weeks (VP-only), while others with birth weight under 1500 g but gestational age over 32 weeks (VLBW-only) form the lower-right quadrant. In Fig. 3, the hexagonal bin plot illustrates the joint distribution of gestational age and birth weight for the POPS study population, with each hexagon’s color reflecting the number of infants in that bin. The majority cluster between approximately 24 and 32 weeks of gestational age and below 1500 g.

Table 1 shows the baseline characteristics at 19, 28 and 35 years of age.^1^ At 19 years, nearly half of participants were both VP & VLBW (n = 320, 49.7%), while approximately 30% were VP–only (n = 192) and 21% were VLBW–only (n = 132). As expected, mean gestational age and birth weight differed significantly across these groups (p < 0.001). The distribution of child sex also varied significantly (p < 0.001), while differences in maternal age at birth were not statistically significant (p = 0.10). At the 28-year follow-up, 47.8% of participants were in the VP & VLBW group (n = 150), while 29.9% were in the VP only group (n = 94) and 22.3% were in the VLBW-only group (n = 70). Gestational age, birth weight, and maternal age at birth differed significantly across these groups (all p < 0.05). At the 35-year follow-up, the cohort consisted of 48.9% VP & VLBW (n = 181), 28.9% VP–only (n = 107), and 22.2% VLBW–only (n = 82). Significant differences between the groups were observed for sex (p = 0.03), gestational age (p < 0.001), birth weight (p < 0.001), and maternal ethnicity (p = 0.03). Medium maternal education level predominated, and the overall ethnic composition was largely Caucasian.

**Table 1:** Baseline characteristics of the POPS cohort by perinatal risk group at 19, 28, and 35 years of age

	VP&VLBW	VP-only	VLBW-only	Total	*p*-value	Missings/ *N* (Pct)
At 19 Years						
*N* (%)	320 (49.7)	192 (29.8)	132 (20.5)	644 (100.0)		0 / 644 (0.00)
Child sex, *N* (%)						
Male	134 (41.9)	82 (42.7)	78 (59.1)	294 (45.7)		
Female	186 (58.1)	110 (57.3)	54 (40.9)	350 (54.3)	$<0.001$	0 / 644 (0.00)
Gestational age (weeks), mean (SD)	29.36 (1.51)	33.97 (1.60)	30.72 (0.97)	31.01 (2.46)	$<0.001$	0 / 644 (0.00)
Birth weight (g), mean (SD)	1161.56 (214.66)	1280.04 (173.99)	1724.02 (198.85)	1312.17 (293.81)	$<0.001$	0/644 (0.00)
Maternal age at birth (years), mean (SD)	27.40 (4.29)	28.21 (5.10)	28.19 (4.85)	27.80 (4.67)	0.10	12 / 644 (1.86)
Maternal education level at birth, *N* (%)						
Low level (ISCED 0–2)	106 (44.9)	38 (29.5)	35 (36.1)	179 (38.7)		
Medium level (ISCED 3–5)	89 (37.7)	62 (48.1)	45 (46.4)	196 (42.4)		
High level (ISCED 6–8)	41 (17.4)	29 (22.5)	17 (17.5)	87 (18.8)	0.06	182 / 644 (28.26)
Maternal ethnicity, *N* (%)						
Caucasian	276 (86.5)	176 (92.6)	117 (90.7)	569 (89.2)		
Non-Caucasian	43 (13.5)	14 (7.4)	12 (9.3)	69 (10.8)	0.08	6 / 644 (0.93)
**At 28 Years**						
* N* (%)	150 (47.8)	94 (29.9)	70 (22.3)	314 (100.0)		0 / 314 (0.00)
Child sex, *N* (%)						
Male	52 (34.7)	32 (34.0)	35 (50.0)	119 (37.9)		
Female	98 (65.3)	62 (66.0)	35 (50.0)	195 (62.1)	0.06	0 / 314 (0.00)
Gestational age (weeks), mean (SD)	29.42 (1.61)	33.72 (1.33)	30.64 (1.04)	30.98 (2.34)	$<0.001$	0 / 314 (0.00)
Birth weight (g), mean (SD)	1147.08 (219.27)	1261.81 (167.40)	1724.29 (209.71)	1310.10 (304.49)	$<0.001$	0/314 (0.00)
Maternal age at birth (years), mean (SD)	27.55 (3.77)	28.57 (4.88)	28.99 (4.56)	28.17 (4.34)	0.04	9 / 314 (2.87)
Maternal education level at birth, *N* (%)						
Low level (ISCED 0–2)	50 (42.7)	24 (31.2)	18 (35.3)	92 (37.6)		
Medium level (ISCED 3–5)	46 (39.3)	35 (45.5)	22 (43.1)	103(42.0)		
High level (ISCED 6–8)	21 (17.9)	18 (23.4)	11 (21.6)	50 (20.4)	0.58	69 / 314 (21.97)
Maternal ethnicity, *N* (%)						
Caucasian	135 (90.0)	91 (97.8)	67 (98.5)	293 (94.2)		
Non-Caucasian	15 (10.0)	2 (2.2)	1 (1.5)	18 (5.8)	0.01	3 / 314 (0.96)
At 35 Years						
*N* (%)	181 (48.9)	107 (28.9)	82 (22.2)	370 (100.0)		0/370 (0.0)
Child sex, *N* (%)						
Male	71 (39.2)	44 (41.1)	46 (56.1)	161 (43.5)		
Female	110 (60.8)	63 (58.9)	36 (43.9)	209 (56.5)	0.03	0/370 (0.0)
Gestational age (weeks), mean (SD)	29.45 (1.56)	33.98 (1.35)	30.75 (0.85)	31.05 (2.37)	< 0.001	0/370 (0.0)
Birth weight (g), mean (SD)	1173.05 (210.41)	1268.22 (162.30)	1733.72 (211.04)	1324.83 (297.19)	< 0.001	0/370 (0.0)
Maternal age at birth (years), mean (SD)	27.87 (3.83)	28.49 (4.36)	28.58 (4.34)	28.21 (4.11)	0.31	8/370 (2.2)
Maternal education level at birth, *N* (%)						
Low level (ISCED 0–2)	54 (38.3)	20 (23.8)	20 (30.3)	94 (32.3)		
Medium level (ISCED 3–5)	61 (43.3)	44 (52.4)	32 (48.5)	137 (47.1)		
High level (ISCED 6–8)	26 (18.4)	20 (23.8)	14 (21.2)	60 (20.6)	0.26	79/370 (21.4)
Maternal ethnicity, *N* (%)						
Caucasian	166 (91.7)	104 (98.1)	76 (97.4)	346 (94.8)		
Non-caucasian	15 (8.3)	2 (1.9)	2 (2.6)	19 (5.2)	0.03	5 / 370 (1.4)

### Distribution of outcome variables by exposures

Table 2 shows that the HUI3 speech domain displayed a significant difference at 19 years (p = 0.01), with suboptimal functioning rates ranging from 8.3% in the VLBW-only group to 20.3% in the VP-only group. Differences in mean and median HUI3 MAU scores utilities have been detected (p = 0.01). Among the 314 individuals assessed at 28 years, ambulation exhibited a notable difference (p < 0.001), driven mainly by an 11.4% suboptimal rate in the VLBW-only group, compared to 2.0% in the VP & VLBW group and 1.1% in the VP-only group. Among 370 participants assessed at 35 years no statistically significant relationships were observed between SF-6D MAU scores and groups considered.

**Table 2:** Proportion with optimal functioning by perinatal risk group at 19, 28, and 35 years of age

Outcomes	VP&VLBW	VP-only	VLBW-only	Total	*p*-value
*HUI3 at 19 years*					
* N* (%)	320 (49.7)	192 (29.8)	132 (20.5)	644 (100.0)	
HUI3-vision optimal functioning	217 (67.8)	114 (59.4)	92 (69.7)	423 (65.7)	0.08
HUI3-hearing optimal functioning	314 (98.1)	189 (98.4)	131 (99.2)	634 (98.4)	0.68
HUI3-speech optimal functioning	264 (82.5)	153 (79.7)	121 (91.7)	538 (83.5)	0.01
HUI3-emotion optimal functioning	211 (65.9)	118 (61.5)	95 (72.0)	424 (65.8)	0.15
HUI3-pain optimal functioning	244 (76.2)	135 (70.3)	104 (78.8)	483 (75.0)	0.17
HUI3-ambulation optimal functioning	316 (98.8)	187 (97.4)	127 (96.2)	630 (97.8)	0.22
HUI3-dexterity optimal functioning	307 (95.9)	184 (95.8)	127 (96.2)	618 (96.0)	0.99
HUI3-cognition optimal functioning	249 (77.8)	134 (69.8)	98 (74.2)	481 (74.7)	0.13
HUI3 MAU score, mean (SD)	0.88 (0.16)	0.84 (0.20)	0.89 (0.18)	0.87 (0.18)	0.01
HUI3 MAU score, median (min; max)	0.93(0.01;1.00)	0.92($-$0.12;1.00)	0.95($-$0.01;1.00)	0.93($-$0.12;1.00)	0.01
*HUI3 at 28 years*					
* N* (%)	150 (47.8)	94 (29.9)	70 (22.3)	314 (100.0)	
HUI3-vision optimal functioning	80 (53.3)	45 (47.9)	39 (55.7)	164 (52.2)	0.57
HUI3-hearing optimal functioning	143 (95.3)	93 (98.9)	69 (98.6)	305 (97.1)	0.19
HUI3-speech optimal functioning	133 (88.7)	82 (87.2)	66 (94.3)	281 (89.5)	0.31
HUI3-emotion optimal functioning	104 (69.3)	69 (73.4)	51 (72.9)	224 (71.3)	0.75
HUI3-pain optimal functioning	113 (75.3)	68 (72.3)	51 (72.9)	232 (73.9)	0.85
HUI3-ambulation optimal functioning	147 (98.0)	93 (98.9)	62 (88.6)	302 (96.2)	<0.001
HUI3-dexterity optimal functioning	146 (97.3)	90 (95.7)	67 (95.7)	303 (96.5)	0.74
HUI3-cognition optimal functioning	119 (79.3)	70 (74.5)	60 (85.7)	249 (79.3)	0.21
HUI3 MAU score, mean (SD)	0.88(0.16)	0.89(0.16)	0.89(0.18)	0.89(0.17)	0.87
HUI3 MAU score, median (min; max)	0.95(0.20;1.00)	0.95(0.22;1.00)	0.97($-$0.05;1.00)	0.96($-$0.05;1.00)	0.49
*SF-6D at 35 years*					
* N* (%)	181 (48.9)	107 (28.9)	82 (22.2)	370 (100.0)	
SF-6D physical optimal functioning	151 (83.4)	88 (82.2)	69 (84.1)	308 (83.2)	0.94
SF-6D role limitations optimal	131 (72.4)	68 (63.6)	61 (74.4)	260 (70.3)	0.19
SF-6D social functioning optimal	95 (52.5)	53 (49.5)	52 (63.4)	200 (54.1)	0.14
SF-6D pain optimal level	127 (70.2)	73 (68.2)	59 (72.0)	259 (70.0)	0.86
SF-6D mental health optimal level	45 (24.9)	23 (21.5)	22 (26.8)	90 (24.3)	0.68
SF-6D vitality optimal level	18 (9.9)	6 (5.6)	5 (6.1)	29 (7.8)	0.33
SF-6D MAU score, mean (SD)	0.81(0.11)	0.79(0.12)	0.81(0.12)	0.81(0.12)	0.18
SF-6D MAU score, median(min;max)	0.86(0.52;1.00)	0.80(0.47;1.00)	0.86(0.54;1.00)	0.86(0.47;1.00)	0.20

*HUI3* Health utility index mark 3, *SF-6D* Short form 6 dimensions, *MAU Scores* Multi-attribute utility scores, *OF* optimal functioning

### Regression results

All regression models in Table 3 used VP & VLBW as the reference group. At 19 years we found that the speech domain showed a significant advantage for the VLBW-only group compared with the VP & VLBW group (*β* = 0*.*11, 95% CI 0*.*02*,*0*.*19, *p* = 0*.*01), indicating a higher likelihood of optimal speech function in this group. At 28 years, domain-level analyses showed mostly null findings, except for two results. First, hearing outcomes were statistically significantly better among VP-only compared with VP & VLBW (*β* = 0*.*05, 95% CI 0*.*00*,* 0*.*10, *p* = 0*.*04), suggesting a better likelihood of optimal hearing if born VP with appropriate weight for gestational age. Second, ambulation outcomes were significantly lower in VLBW-only relative to the reference group (*β* =  − 0*.*12, 95% CI − 0*.*18*,* − 0*.*05, *p* < 0*.0*01). We found a borderline association for speech in VLBW-only (*β* = 0*.*09, *p* = 0*.*09). There were no statistically significant differences in any of the SF-6D domains or MAU scores between VP & VLBW group and VLBW-only or VP-only. Table A.2 in appendix presents results of the logistic regression models adjusted for covariates which are consistent with those reported in Table 3.

**Table 3:** Adjusted regression analysis by VP, VLBW status

		Coefficient	SE(Coeff.)	Lower 95% CI	Upper 95% CI	*p*-value
*HUI3 outcomes at 19 years*						
HUI3-MAU scores	$\beta _{\text {VP-only} }$	$-$0.03	0.02	$-$0.06	0.01	0.17
	$\beta _{\text {VLBW-only} }$	0.02	0.02	$-$0.02	0.06	0.45
HUI3-vision OF	$\beta _{\text {VP-only} }$	$-$0.04	0.05	$-$0.15	0.06	0.42
	$\beta _{\text {VLBW-only} }$	0.00	0.06	$-$0.11	0.12	0.94
HUI3-hearing OF	$\beta _{\text {VP-only} }$	$-$0.00	0.01	$-$0.03	0.03	0.98
	$\beta _{\text {VLBW-only} }$	0.01	0.01	$-$0.01	0.04	0.35
HUI3-speech OF	$\beta _{\text {VP-only} }$	$-$0.01	0.04	$-$0.08	0.06	0.78
	$\beta _{\text {VLBW-only} }$	0.11**	0.04	0.02	0.19	0.01
HUI3-emotion OF	$\beta _{\text {VP-only} }$	$-$0.03	0.05	$-$0.14	0.07	0.54
	$\beta _{\text {VLBW-only} }$	0.02	0.06	$-$0.09	0.14	0.70
HUI3-pain OF	$\beta _{\text {VP-only} }$	0.01	0.05	$-$0.08	0.10	0.87
	$\beta _{\text {VLBW-only} }$	0.05	0.05	$-$0.05	0.15	0.29
HUI3-ambulation OF	$\beta _{\text {VP-only} }$	0.00	0.02	$-$0.03	0.03	0.91
	$\beta _{\text {VLBW-only} }$	$-$0.01	0.02	$-$0.05	0.02	0.49
HUI3-dexterity OF	$\beta _{\text {VP-only} }$	$-$0.01	0.02	$-$0.05	0.04	0.76
	$\beta _{\text {VLBW-only} }$	0.01	0.02	$-$0.04	0.06	0.71
HUI3-cognition OF	$\beta _{\text {VP-only} }$	$-$0.06	0.05	$-$0.15	0.04	0.23
	$\beta _{\text {VLBW-only} }$	$-$0.05	0.05	$-$0.15	0.05	0.31
*HUI3 outcomes at 28 years*						
HUI3-MAU Scores	$\beta _{\text {VP-only} }$	0.03	0.03	$-$0.02	0.08	0.31
	$\beta _{\text {VLBW-only} }$	0.02	0.03	$-$0.03	0.08	0.44
HUI3-vision OF	$\beta _{\text {VP-only} }$	0.01	0.08	$-$0.14	0.16	0.87
	$\beta _{\text {VLBW-only} }$	0.05	0.09	$-$0.12	0.22	0.59
HUI3-hearing OF	$\beta _{\text {VP-only} }$	0.05**	0.03	0.00	0.10	0.04
	$\beta _{\text {VLBW-only} }$	0.04	0.03	$-$0.02	0.10	0.20
HUI3-speech OF	$\beta _{\text {VP-only} }$	0.06	0.05	$-$0.03	0.16	0.18
	$\beta _{\text {VLBW-only} }$	0.09*	0.05	$-$0.01	0.20	0.09
HUI3-emotion OF	$\beta _{\text {VP-only} }$	0.08	0.07	$-$0.05	0.21	0.23
	$\beta _{\text {VLBW-only} }$	0.01	0.08	$-$0.14	0.17	0.86
HUI3-pain OF	$\beta _{\text {VP-only} }$	0.01	0.07	$-$0.12	0.14	0.88
	$\beta _{\text {VLBW-only} }$	$-$0.01	0.08	$-$0.16	0.13	0.85
HUI3-ambulation OF	$\beta _{\text {VP-only} }$	$-$0.01	0.03	$-$0.06	0.05	0.84
	$\beta _{\text {VLBW-only} }$	$-$0.12***	0.03	$-$0.18	$-$0.05	<0.001
HUI3-dexterity OF	$\beta _{\text {VP-only} }$	$-$0.01	0.03	$-$0.07	0.04	0.62
	$\beta _{\text {VLBW-only} }$	$-$0.02	0.03	$-$0.09	0.05	0.54
HUI3-cognition OF	$\beta _{\text {VP-only} }$	0.02	0.06	$-$0.10	0.15	0.69
	$\beta _{\text {VLBW-only} }$	0.09	0.07	$-$0.05	0.23	0.20
*SF-6D outcomes at 35 years*						
SF-6D MAU Scores	$\beta _{\text {VP-only} }$	$-$0.02	0.02	$-$0.05	0.02	0.32
	$\beta _{\text {VLBW-only} }$	$-$0.00	0.02	$-$0.04	0.03	0.97
SF-6D physical OF	$\beta _{\text {VP-only} }$	$-$0.01	0.05	$-$0.11	0.10	0.88
	$\beta _{\text {VLBW-only} }$	$-$0.01	0.06	$-$0.12	0.11	0.88
SF-6D role limitations OF	$\beta _{\text {VP-only} }$	$-$0.09	0.06	$-$0.22	0.04	0.16
	$\beta _{\text {VLBW-only} }$	0.01	0.07	$-$0.13	0.15	0.86
SF-6D social OF	$\beta _{\text {VP-only} }$	0.04	0.07	$-$0.09	0.18	0.53
	$\beta _{\text {VLBW-only} }$	0.11	0.08	$-$0.04	0.26	0.14
SF-6D pain OF	$\beta _{\text {VP-only} }$	0.02	0.06	$-$0.11	0.15	0.74
	$\beta _{\text {VLBW-only} }$	0.04	0.07	$-$0.10	0.18	0.59
SF-6D mental health OF	$\beta _{\text {VP-only} }$	$-$0.04	0.06	$-$0.16	0.08	0.52
	$\beta _{\text {VLBW-only} }$	0.02	0.07	$-$0.11	0.16	0.73
SF-6D vitality OF	$\beta _{\text {VP-only} }$	$-$0.03	0.04	$-$0.10	0.04	0.40
	$\beta _{\text {VLBW-only} }$	$-$0.05	0.04	$-$0.12	0.03	0.25

***p < 0.001; **p < 0.05; *p < 0.1

*HUI3* Health utility index mark 3, *SF-6D* short form 6 dimensions, *MAU Scores* multi-attribute utility scores, *OF* optimal functioning. All models adjusted for: sex, age (in years) at assessment, mother’s level of education, maternal age and maternal ethnicity

The attrition–adjusted model applied IPW that were estimated from a logistic regression predicting whether an individual remained in the study at age 28 and 35 years (results in Table A.3). The coefficients and statistical significance of some key findings were virtually identical after accounting for attrition. In the unweighted model at 28 years, the VP-only group had significantly better hearing (*β* = 0*.*05*, p* = 0*.*04); this relationship became also significant in the weighted model (*β* = 0*.*05*, p* = 0.02). Similarly, the significant ambulation deficit in the VLBW-only group (*β* =  − 0*.*12*, p* < 0*.*001) and the same relationship have been found for the VLBW-only group (*β* =  − 0*.*14*, p* = 0*.*02) in the weighted model. No statistical relationships have been found in the weighted models at 35 years.

Several maternal and demographic covariates emerged as strong predictors of HRQoL at all timepoints considered Table A.4. Female sex was associated with significantly different health outcomes at each time point, showing a pattern of worsening outcomes in specific physical and mental domains over time. At age 19, being female was associated with a lower probability of optimal functioning in vision (β = − 0.117, p = 0.010) and pain (β = − 0.121, p = 0.002), but a higher probability of optimal cognitive functioning (β = 0.103, p = 0.010). At 28, the association with a lower probability of optimal vision functioning persisted (β = − 0.146, p = 0.031). By age 35, being female was consistently linked to poorer outcomes, including a lower overall SF-6D utility score (β = − 0.038, p = 0.007) and a lower probability of optimal functioning in social (β = − 0.174, p = 0.004), pain (β = − 0.113, p = 0.042), and mental health (β = − 0.171, p = 0.001) domains.

In contrast, other demographic factors showed less consistent patterns over time. Maternal age did not have a consistent impact; a small, statistically significant positive association with the overall HUI3 utility score at age 19 (β = 0.005, p < 0.05) did not remain significant at ages 28 or 35. The effect of ethnicity appeared to become significant only in later adulthood. While there were no significant differences for participants born to non-Caucasian mothers at ages 19 and 28, they reported significantly better social functioning at age 35 (β = 0.329, p < 0.05).

We have examined the adjusted regression coefficients on outcomes stratified by sex in Table A.5. Our analysis, stratified by sex, reveals distinct HRQoL outcomes by sex. For males, at age 19, the VP-only group had a higher probability of optimal speech (*β* = 0*.*12, p = 0.03) while the VLBW-only group had a lower probability of optimal emotion (*β* =  − 0*.*17, p = 0.03). At age 28, the VLBW-only group showed an advantage in optimal speech functioning (*β* = 0*.*19, p = 0.04), whereas the VP-only group was associated with a lower probability of optimal ambulation functioning (*β* =  − 0*.*17, p < 0.001). In contrast, no significant differences were observed for females at ages 19 or 28. However, by age 35, female sex in the VLBW-only group was associated with significantly lower SF-6D utility scores (*β* =  − 0*.*05, p = 0.01) and poorer outcomes in role functioning (*β* =  − 0*.*20, p = 0.02) and vitality (*β* =  − 0*.*09, p = 0.03), with the VP-only group also showing a decrease in vitality domain (*β* =  − 0*.*11, p = 0.03).

### Discussion

To the best of our knowledge, we are the first to report the differential impact of VP vs. VLBW— separately and together—on early and mid-adult domain-level HRQoL. Overall, results show that those born VP & VLBW do not appear to have lower MAU scores than VP-only or VLBW-only, however, significant differences were identify across specific domains at 19 and 28 when outcomes were measured with HUI3. In particular, the domain-specific advantages we detected (speech at 19 years for VLBW-only and hearing at 28 years for VP-only) and the deficit (ambulation at 28 years for VLBW-only) constitute a fresh contribution to the literature. No significant deficits were observed at 35 years follow up. Attrition-weighted analyses did not change the results materially after IPW adjustment.

At 19 years, female sex predicted worse vision and pain but better cognition. Main effects for sex were also significant at ages 28 and 35, and sex-stratified results are presented for all ages. Female sex remained associated with lower vision at 28 years and lower overall utility, social functioning, pain and mental health at 35 years. This sex difference may have implications for targeted resource allocation and could stem from both biological and social factors. Finally, increasing maternal age was related to small but positive shifts in overall HRQoL, consistent with evidence that older maternal age can confer socioeconomic or experiential advantages. These results accord with prior research demonstrating that high SES can be protective among both VP/VLBW and term-born individuals [47, 48].

Our findings align with earlier POPS publications showing a high risk of neonatal complications in the VP & VLBW group. While we observed a convergence in overall HRQoL scores by mid-adulthood, specific deficits persisted at 19 and 28 years. This suggests that while some catch-up in general well-being may occur, the long-term sequelae of immaturity versus fetal growth restriction follow distinct pathways. Previous POPS studies documented persistent differences in growth parameters (e.g., height, weight, BMI) [27, 49] and some neurodevelopmental deficits at 19 years, yet these do not always translate into overall HRQoL deficits at 19, 28, or 35 years. Our data thus confirm that some subgroups experience meaningful improvement over time.

This study emphasizes that VP and VLBW categories are not interchangeable, which has important implications for healthcare resource allocation. Our approach offers new insights that go beyond evaluating VP/VLBW as a single, homogenous category. The unique outcomes associated with each category indicate that insights from VLBW-focused studies may not seamlessly apply to VP populations, and vice versa. Given this distinction, healthcare resources may need to be allocated differently depending on whether a neonate is VP, VLBW, or both, ensuring more efficient use of funds and improved health outcomes. Moreover, the cost-effectiveness of interventions related to preterm birth can vary by category. Domain-specific interventions, such as addressing hearing or dexterity issues, may optimize resource use. Future research should explore cost-effectiveness analyses and targeted early intervention programs to improve long-term HRQoL in those born at substantially lower gestational ages. Furthermore, our study shows that incremental improvements in MAU-scores from 19 to 35 years in some subgroups suggest that early deficits may be partially remediable over time, though not uniformly so across the cohort. These hypotheses warrant further investigation to elucidate the mechanisms driving observed long-term outcomes.

One strength of this study is that POPS achieved approximately 94% enrollment of all VP/VLBW infants born in 1983, reducing selection bias and bolstering the internal validity of our findings. Additionally, our focus on both the combined and distinct impacts of VP and VLBW goes beyond treating these categories as homogeneous. Nevertheless, a substantial proportion of participants were lost due to mortality or other factors, potentially introducing attrition bias. Caution is therefore warranted in interpreting the long-term HRQoL results. Furthermore, some subgroups, particularly when stratified into VP-only and VLBW-only, have relatively small sample sizes. This raises concerns about the power to detect meaningful differences. Because 35 years HUI3 has been replaced with SF-6D this prevents a homogeneous comparability between results at 35 years with earlier follow up time points.

We acknowledge that data on family income around birth was not collected in the POPS cohort. Thus, our proxies to control for socioeconomic status around birth such as maternal education, maternal age and maternal ethnicity [42–46] might not fully reduce confounding from early-life socioeconomic disadvantage. Finally, as this study took place in a single high-income country with advanced neonatal care, generalizability to lower- or middle-income settings may be limited. Different healthcare infrastructure, socioeconomic contexts, or population characteristics may alter these long-term trajectories.

## Conclusion

In this national cohort, VP & VLBW status yielded no consistent differences in overall multi-attribute utility scores by 35 years, yet domain-specific disparities such as speech advantage for VLBW-only, ambulation deficit for VP-only were evident at 19 and 28 years and varied by sex. Because outcomes at 35 years were measured with SF-6D rather than HUI3, the cross-wave comparisons must be interpreted cautiously.

Overall, results suggest that long-term follow-up programs should be tailored to domain-specific risks rather than a homogenous ‘preterm’ label. Our findings underscore that VP and VLBW are distinct risk categories which might require tailored, domain-focused interventions. Future studies should use consistent HRQoL measurement tools to identify whether the observed convergence is sustained and how sex-specific vulnerabilities evolve over the life-course trajectories.

## Supplementary Information

Below is the link to the electronic supplementary material.

Supplementary file1 (PDF 518 KB)

## Data Availability

The data that support the findings of this study are not available due to restrictions related to participant privacy and the informed consent under which the data were collected.

## Footnotes

1 Table A.1 in the Appendix for the same characteristics presented wave-by-wave.
