# Supplementary material for: The impact of very preterm vs very low birth weight on early and mid-adulthood preference-based HRQoL outcomes: findings from the Dutch study on preterm and small for gestational age infants
Source: Qual Life Res. 2025 Sep 13;34(12):3511–23. doi: 10.1007/s11136-025-04024-8 (PMC12689665; doi:10.1007/s11136-025-04024-8)
Supplement: Supplementary file 1 — Supplementary file1 (PDF 518 KB) [file 11136_2025_4024_MOESM1_ESM.pdf]

A Appendix

Table A.1: Baseline Characteristics of Participants at Each Follow-up Wave

**Characteristic**	**At 19 Years** (n=644)	**At 28 Years** (n=314)	**At 35 Years** (n=370)
**Child Sex, n (%)**			
Male	294 (45.7)	119 (37.9)	161 (43.5)
Female	350 (54.3)	195 (62.1)	209 (56.5)
**Neonatal Characteristics, mean (SD)**			
Gestational age (weeks)	31.01 (2.46)	30.98 (2.34)	31.05 (2.37)
Birth weight (grams)	1312.17 (293.81)	1310.10 (304.49)	1324.83 (297.19)
**Maternal Characteristics**			
Age at birth (years), mean (SD)	27.80 (4.67)	28.17 (4.34)	28.21 (4.11)
Missing, n (%)	*12 (1.9)*	*9 (2.9)*	*8 (2.2)*
**Maternal Education, n (%)**			
Low level (ISCED 0–2)	179 (38.7)	92 (37.6)	94 (32.3)
Medium level (ISCED 3–5)	196 (42.4)	103 (42.0)	137 (47.1)
High level (ISCED 6–8)	87 (18.8)	50 (20.4)	60 (20.6)
Missing, n (%)	*182 (28.3)*	*69 (22.0)*	*79 (21.4)*
**Maternal Ethnicity, n (%)**			
Caucasian	569 (89.2)	293 (94.2)	346 (94.8)
Non-caucasian	69 (10.8)	18 (5.8)	19 (5.2)
Missing, n (%)	*6 (0.9)*	*3 (1.0)*	*5 (1.4)*

Notes: Data are presented as n (%) for categorical variables and mean (SD) for continuous variables. The sample at each wave consists of all participants with a valid HRQoL score at that specific age.

Table A.2: Adjusted Odds Ratios for Optimal Health-Related Quality of Life Outcomes by Neonatal Risk Group

Outcome	Exposure Group (vs. VP & VLBW)	Odds Ratio (OR)	Std. Error (SE)	Lower 95% CI	Upper 95% CI	*p*-value
**Panel A: HUI3 Outcomes at 19 Years**						
**Optimal Vision**	VLBW-only (GA≥32, BW<1500g)	0.83	1.27	0.52	1.32	0.43
	VP-only (GA<32, BW≥1500g)	1.03	1.31	0.60	1.75	0.92
**Optimal Hearing**	VLBW-only	1.02	2.51	0.17	6.18	0.98
**Optimal Speech**	VLBW-only	0.89	1.38	0.47	1.68	0.72
	VP-only	**3.98****	**1.73**	**1.36**	**11.69**	**0.01**
**Optimal Emotion**	VLBW-only	0.86	1.27	0.54	1.38	0.54
	VP-only	1.11	1.31	0.65	1.90	0.70
**Optimal Pain**	VLBW-only	1.06	1.31	0.62	1.80	0.84
	VP-only	1.46	1.39	0.76	2.78	0.25
**Optimal Ambulation**	VLBW-only	1.17	2.44	0.20	6.71	0.86
	VP-only	0.63	2.21	0.13	2.98	0.56
**Optimal Dexterity**	VLBW-only	0.82	1.79	0.26	2.57	0.73
	VP-only	1.32	1.99	0.34	5.10	0.69
**Optimal Cognition**	VLBW-only	0.71	1.32	0.41	1.22	0.22
	VP-only	0.74	1.35	0.41	1.32	0.31
**Panel B: HUI3 Outcomes at 28 Years**						
**Optimal Vision**	VLBW-only	1.06	1.36	0.58	1.94	0.86
	VP-only	1.22	1.43	0.60	2.46	0.58
**Optimal Hearing**	VP-only	3.20	3.04	0.36	28.24	0.29
**Optimal Speech**	VLBW-only	1.91	1.67	0.70	5.19	0.21
	VP-only	2.83	1.94	0.77	10.35	0.12
**Optimal Emotion**	VLBW-only	1.56	1.43	0.77	3.17	0.22
	VP-only	1.07	1.47	0.50	2.29	0.86
**Optimal Pain**	VLBW-only	1.06	1.43	0.53	2.13	0.87
	VP-only	0.93	1.49	0.42	2.03	0.85
**Optimal Ambulation**	VLBW-only	0.67	4.09	0.04	10.61	0.78
	VP-only	**0.06****	**3.03**	**0.01**	**0.52**	**0.01**
**Optimal Dexterity**	VLBW-only	0.58	2.34	0.11	3.07	0.52
	VP-only	0.49	2.64	0.07	3.28	0.46
**Optimal Cognition**	VLBW-only	1.16	1.44	0.57	2.38	0.69
	VP-only	1.87	1.61	0.74	4.75	0.19
**Panel C: SF-6D Outcomes at 35 Years**						
**Optimal Physical Func.**	VLBW-only	0.94	1.46	0.45	1.98	0.87
	VP-only	0.94	1.53	0.41	2.15	0.88
**Optimal Role Func.**	VLBW-only	0.65	1.36	0.36	1.18	0.16
	VP-only	1.07	1.42	0.53	2.14	0.85
**Optimal Social Func.**	VLBW-only	1.20	1.34	0.68	2.11	0.53
	VP-only	1.62	1.38	0.86	3.06	0.13
**Optimal Pain**	VLBW-only	1.11	1.37	0.60	2.04	0.74
	VP-only	1.22	1.42	0.61	2.42	0.58
**Optimal Mental Health**	VLBW-only	0.80	1.40	0.41	1.55	0.50
	VP-only	1.12	1.42	0.57	2.21	0.74
**Optimal Vitality**	VLBW-only	0.62	1.75	0.21	1.87	0.40
	VP-only	0.47	1.96	0.13	1.75	0.26

Notes: Results are Odds Ratios (OR) from adjusted logistic regression models. The reference group is VP & VLBW. VLBW-only: Gestational Age ≥32 weeks & Birth Weight <1500g. VP-only: Gestational Age <32 weeks & Birth Weight ≥1500g. All models adjusted for sex, maternal age, maternal education, and maternal ethnicity. ** indicates p<0.05.

Table A.3: Inverse Probability Weighted Regression Models for HRQoL Outcomes at 28 and 35 Years

Outcome	Exposure Group (vs. VP & VLBW)	Coefficient (β)	Std. Error (SE)	Lower 95% CI	Upper 95% CI	*p*-value
**Panel A: HUI3 Outcomes at 28 Years (n=237)**						
**HUI3-MAU Score**	VLBW-only	0.02	0.03	-0.04	0.07	0.54
	VP-only	0.01	0.04	-0.07	0.09	0.86
**Optimal Vision**	VLBW-only	0.03	0.08	-0.13	0.18	0.74
	VP-only	0.04	0.09	-0.14	0.22	0.65
**Optimal Hearing**	VLBW-only	**0.06****	**0.02**	**0.01**	**0.11**	**0.02**
	VP-only	0.04	0.04	-0.03	0.11	0.26
**Optimal Speech**	VLBW-only	0.08	0.05	-0.02	0.17	0.12
	VP-only	0.09	0.06	-0.04	0.21	0.17
**Optimal Emotion**	VLBW-only	0.05	0.07	-0.09	0.19	0.50
	VP-only	0.00	0.09	-0.17	0.18	0.97
**Optimal Pain**	VLBW-only	-0.01	0.07	-0.15	0.13	0.91
	VP-only	-0.01	0.08	-0.17	0.15	0.89
**Optimal Ambulation**	VLBW-only	-0.00	0.02	-0.03	0.03	0.88
	VP-only	**-0.14****	**0.06**	**-0.25**	**-0.03**	**0.02**
**Optimal Dexterity**	VLBW-only	-0.01	0.03	-0.07	0.05	0.71
	VP-only	-0.03	0.04	-0.11	0.06	0.54
**Optimal Cognition**	VLBW-only	0.00	0.07	-0.13	0.14	0.98
	VP-only	0.08	0.07	-0.07	0.22	0.31
**Panel B: SF-6D Outcomes at 35 Years (n=282)**						
**SF-6D MAU Score**	VLBW-only	-0.01	0.02	-0.05	0.02	0.43
	VP-only	0.00	0.02	-0.04	0.04	0.98
**Optimal Physical Func.**	VLBW-only	-0.01	0.06	-0.12	0.10	0.83
	VP-only	0.01	0.06	-0.10	0.12	0.87
**Optimal Role Func.**	VLBW-only	-0.08	0.07	-0.21	0.06	0.26
	VP-only	0.03	0.07	-0.11	0.16	0.72
**Optimal Social Func.**	VLBW-only	0.06	0.07	-0.09	0.20	0.45
	VP-only	0.13	0.08	-0.02	0.29	0.09
**Optimal Pain**	VLBW-only	0.03	0.07	-0.10	0.17	0.64
	VP-only	0.06	0.07	-0.09	0.20	0.45
**Optimal Mental Health**	VLBW-only	-0.02	0.06	-0.14	0.10	0.74
	VP-only	0.02	0.07	-0.12	0.15	0.81
**Optimal Vitality**	VLBW-only	-0.04	0.04	-0.12	0.04	0.32
	VP-only	-0.06	0.04	-0.13	0.01	0.10

Notes: Models are adjusted for sex, maternal age, maternal education, and maternal ethnicity. The reference group is VP & VLBW. VLBW-only: Gestational Age $\geq$32 weeks & Birth Weight $<$1500g. VP-only: Gestational Age $<$32 weeks & Birth Weight $\geq$1500g. OF denotes Optimal Functioning. Coefficients are from linear probability models. ** indicates p$<$0.05. Coefficients are from linear probability models. All models are weighted using inverse probability weighting to adjust for attrition.

Table A.4: Full Adjusted Regression Models for HRQoL Outcomes at Ages 19, 28, and 35 Years

	Panel A: HUI3 Outcomes at 19 Years								
Variable	HUI3 MAU	Vision OF	Hearing OF	Speech OF	Emotion OF	Pain OF	Amb. OF	Dext. OF	Cogn. OF
VLBW-only	−0.025	−0.043	0.000	−0.011	−0.032	0.007	0.002	−0.007	−0.056
	(0.171)	(0.421)	(0.976)	(0.776)	(0.540)	(0.874)	(0.906)	(0.764)	(0.233)
VP-only	0.016	0.004	0.013	0.106**	0.022	0.054	−0.012	0.009	−0.052
	(0.446)	(0.940)	(0.350)	(0.011)	(0.703)	(0.290)	(0.492)	(0.706)	(0.308)
Female	0.004	−0.117**	−0.015	0.019	−0.003	−0.121**	0.012	0.021	0.103**
	(0.813)	(0.010)	(0.181)	(0.555)	(0.947)	(0.002)	(0.355)	(0.250)	(0.010)
Maternal Age	0.005**	0.003	0.002	0.006	0.000	0.009	0.002	0.002	0.008
	(0.009)	(0.561)	(0.175)	(0.144)	(0.951)	(0.056)	(0.279)	(0.338)	(0.080)
Maternal Educ. (Med)	−0.018	−0.075	−0.001	0.061	−0.040	−0.023	−0.025	0.000	0.018
	(0.313)	(0.133)	(0.954)	(0.086)	(0.419)	(0.602)	(0.097)	(0.991)	(0.685)
Maternal Educ. (High)	−0.025	−0.175**	0.010	0.073	−0.180**	−0.047	0.005	0.012	−0.051
	(0.272)	(0.008)	(0.551)	(0.118)	(0.006)	(0.411)	(0.814)	(0.666)	(0.375)
Non-Caucasian	−0.016	0.041	0.012	−0.073	−0.140	0.128	0.018	−0.043	−0.094
	(0.609)	(0.662)	(0.582)	(0.267)	(0.127)	(0.115)	(0.507)	(0.258)	(0.245)

	Panel B: HUI3 Outcomes at 28 Years								
Variable	HUI3 MAU	Vision OF	Hearing OF	Speech OF	Emotion OF	Pain OF	Ambulation OF	Dexterity OF	Cognition OF
VLBW-only	0.026	0.013	0.052**	0.063	0.081	0.010	−0.006	−0.014	0.025
	(0.312)	(0.866)	(0.043)	(0.177)	(0.232)	(0.880)	(0.842)	(0.625)	(0.691)
VP-only	0.023	0.047	0.038	0.092	0.013	−0.015	−0.117**	−0.020	0.093
	(0.437)	(0.591)	(0.199)	(0.087)	(0.864)	(0.845)	(0.000)	(0.541)	(0.197)
Female	0.000	−0.146**	0.040	0.042	0.049	−0.044	0.001	−0.023	−0.030
	(0.987)	(0.031)	(0.085)	(0.313)	(0.417)	(0.454)	(0.958)	(0.382)	(0.594)
Maternal Age	0.000	0.003	0.002	−0.002	−0.013	−0.007	0.005	0.005	−0.006
	(0.971)	(0.709)	(0.477)	(0.653)	(0.064)	(0.319)	(0.130)	(0.118)	(0.327)
Maternal Educ. (Med)	0.006	−0.090	−0.020	−0.015	−0.018	0.083	−0.013	0.016	0.035
	(0.807)	(0.215)	(0.412)	(0.735)	(0.787)	(0.195)	(0.638)	(0.568)	(0.566)
Maternal Educ. (High)	−0.011	−0.161	−0.012	0.044	−0.160**	0.040	0.004	−0.008	0.067
	(0.727)	(0.080)	(0.705)	(0.439)	(0.050)	(0.617)	(0.903)	(0.832)	(0.376)
Non-Caucasian	−0.030	0.229	0.039	0.130	−0.025	0.116	−0.131	−0.107	−0.189
	(0.642)	(0.237)	(0.556)	(0.277)	(0.882)	(0.496)	(0.073)	(0.154)	(0.240)

	Panel D: SF-6D Outcomes at 35 Years						
Variable	SF-6D Utility	Physical OF	Role OF	Social OF	Pain OF	Mental OF	Vitality OF
VLBW-only	−0.016	−0.008	−0.091	0.043	0.021	−0.040	−0.030
	(0.322)	(0.878)	(0.162)	(0.531)	(0.744)	(0.518)	(0.404)
VP-only	−0.001	−0.008	0.013	0.113	0.038	0.024	−0.046
	(0.967)	(0.884)	(0.857)	(0.138)	(0.589)	(0.727)	(0.247)
Female	−0.038**	−0.067	−0.067	−0.174**	−0.113**	−0.171**	−0.031
	(0.007)	(0.141)	(0.231)	(0.004)	(0.042)	(0.001)	(0.325)
Maternal Age	0.002	0.008	0.005	0.003	0.003	−0.002	0.000
	(0.277)	(0.135)	(0.450)	(0.731)	(0.629)	(0.764)	(0.963)
Maternal Educ. (Med)	0.026	0.037	0.135**	0.011	−0.029	−0.005	0.045
	(0.108)	(0.472)	(0.033)	(0.868)	(0.640)	(0.931)	(0.207)
Maternal Educ. (High)	0.005	−0.007	0.086	−0.080	−0.053	−0.023	−0.016
	(0.790)	(0.916)	(0.280)	(0.348)	(0.504)	(0.763)	(0.717)
Non-Caucasian	0.038	0.091	0.120	0.329**	0.132	0.077	−0.076
	(0.313)	(0.454)	(0.422)	(0.040)	(0.373)	(0.586)	(0.363)

Notes: Reference group for exposures is VP & VLBW. All models adjusted for all other covariates listed in the table. p-values are in parentheses below coefficients. OF denotes Optimal Functioning. Coefficients are from linear probability models. Results for ages 28 and 35 are presented from unweighted models for direct comparison with the age 19 results. ** indicates p<0.05.

Table A.5: Adjusted Regression Coefficients for HRQoL Outcomes by Neonatal Risk Group, Stratified by Sex

Outcome	Exposure vs. VP & VLBW	Males Coefficient	Males (p-value)	Females Coefficient	Females (p-value)
**Panel A: HUI3 Outcomes at 19 Years**					
**HUI3-MAU Score**	VLBW-only	-0.01	(0.80)	-0.04	(0.11)
	VP-only	0.01	(0.68)	0.03	(0.36)
**Optimal Vision**	VLBW-only	0.05	(0.49)	-0.11	(0.12)
	VP-only	0.11	(0.15)	-0.12	(0.22)
**Optimal Hearing**	VLBW-only	0.01	(0.45)	-0.01	(0.73)
	VP-only	0.01	(0.42)	0.02	(0.47)
**Optimal Speech**	VLBW-only	0.02	(0.78)	-0.03	(0.57)
	VP-only	**0.12****	**(0.03)**	0.09	(0.14)
**Optimal Emotion**	VLBW-only	**-0.17****	**(0.03)**	0.07	(0.34)
	VP-only	0.01	(0.85)	0.02	(0.80)
**Optimal Pain**	VLBW-only	-0.02	(0.72)	0.03	(0.61)
	VP-only	0.04	(0.56)	0.08	(0.33)
**Optimal Ambulation**	VLBW-only	0.01	(0.80)	-0.00	(0.91)
	VP-only	-0.01	(0.78)	-0.02	(0.46)
**Optimal Dexterity**	VLBW-only	0.01	(0.75)	-0.02	(0.48)
	VP-only	0.02	(0.54)	-0.01	(0.85)
**Optimal Cognition**	VLBW-only	-0.08	(0.29)	-0.03	(0.59)
	VP-only	-0.05	(0.53)	-0.05	(0.50)
**Panel B: HUI3 Outcomes at 28 Years**					
**HUI3-MAU Score**	VLBW-only	0.06	(0.15)	0.01	(0.65)
	VP-only	0.02	(0.66)	0.04	(0.36)
**Optimal Vision**	VLBW-only	0.10	(0.44)	-0.04	(0.65)
	VP-only	0.08	(0.53)	-0.00	(0.98)
**Optimal Hearing**	VLBW-only	0.10	(0.10)	0.03	(0.20)
	VP-only	0.05	(0.44)	0.03	(0.33)
**Optimal Speech**	VLBW-only	**0.19****	**(0.04)**	0.01	(0.87)
	VP-only	0.12	(0.17)	0.07	(0.33)
**Optimal Emotion**	VLBW-only	0.13	(0.28)	0.06	(0.46)
	VP-only	0.12	(0.33)	-0.06	(0.57)
**Optimal Pain**	VLBW-only	0.11	(0.31)	-0.02	(0.85)
	VP-only	0.07	(0.49)	-0.05	(0.63)
**Optimal Ambulation**	VLBW-only	-0.00	(0.95)	-0.01	(0.84)
	VP-only	**-0.17*****	**(< 0.001)**	-0.06	(0.15)
**Optimal Dexterity**	VLBW-only	0.02	(0.67)	-0.03	(0.44)
	VP-only	-0.01	(0.74)	-0.01	(0.81)
**Optimal Cognition**	VLBW-only	0.10	(0.31)	0.00	(0.97)
	VP-only	0.12	(0.25)	0.08	(0.43)
**Panel C: SF-6D Outcomes at 35 Years**					
**SF-6D Utility Score**	VLBW-only	0.03	(0.17)	**-0.05****	**(0.01)**
	VP-only	0.03	(0.24)	-0.03	(0.23)
**Optimal Physical Func.**	VLBW-only	0.08	(0.29)	-0.07	(0.35)
	VP-only	-0.04	(0.55)	0.03	(0.74)
**Optimal Role Func.**	VLBW-only	0.06	(0.55)	**-0.20****	**(0.02)**
	VP-only	0.09	(0.38)	-0.07	(0.47)
**Optimal Social Func.**	VLBW-only	0.17	(0.11)	-0.04	(0.63)
	VP-only	0.13	(0.22)	0.10	(0.37)
**Optimal Pain**	VLBW-only	0.07	(0.45)	-0.02	(0.86)
	VP-only	-0.00	(0.97)	0.07	(0.49)
**Optimal Mental Health**	VLBW-only	-0.01	(0.90)	-0.07	(0.32)
	VP-only	0.13	(0.22)	-0.09	(0.33)
**Optimal Vitality**	VLBW-only	0.05	(0.42)	**-0.09****	**(0.03)**
	VP-only	0.02	(0.72)	**-0.11****	**(0.03)**

Notes: Models are adjusted for maternal age, maternal education, and maternal ethnicity. The reference group is VP & VLBW. VLBW-only: Gestational Age $\geq$32 weeks & Birth Weight $<$1500g. VP-only: Gestational Age $<$32 weeks & Birth Weight $\geq$1500g. OF denotes Optimal Functioning. Coefficients are from linear probability models. p-values are in parentheses. Statistically significant results ($p<0.05$) are highlighted in bold. Results for ages 28 and 35 are from models using inverse probability weighting (IPW) to adjust for attrition.
